# Computational Fluid Dynamics Modeling of Pressure-Retarded Osmosis: Towards a Virtual Lab for Osmotic-Driven Process Simulations

**DOI:** 10.3390/membranes14110236

**Published:** 2024-11-11

**Authors:** Meisam Mohammadi Amin, Ulrich Krühne

**Affiliations:** Process and Systems Engineering Centre (PROSYS), Technical University of Denmark (DTU), DK-2800 Lyngby, Denmark; ulkr@kt.dtu.dk

**Keywords:** CFD, FO, membrane, osmotic energy, pressure-retarded osmosis (PRO), virtual lab

## Abstract

Pressure-Retarded Osmosis (PRO) is an osmotically driven membrane-based process that has recently garnered significant attention from researchers due to its potential for clean energy harvesting from salinity gradients. The complex interactions between mixed-mode channel flows and osmotic fluxes in real PRO membrane modules necessitate high-fidelity modeling approaches. In this work, an efficient CFD framework is developed for the 3D simulation of osmotically driven membrane processes. This approach is based on a two-way coupling between a CFD solver, which captures external concentration polarization (ECP) effects, and an analytical representation of internal concentration polarization (ICP). Consequently, the osmotic water flux and reverse salt flux (RSF) can be accurately determined, accounting for all CP effects without any limitations on the geometrical complexity of the membrane chamber or its flow mode/regime. The proposed model is validated against experimental data, showing good agreement across various PRO case studies. Additionally, the model’s flexibility to simulate other types of osmotically driven processes such as forward osmosis (FO) is examined. Thus, the contributions of ECP and ICP effects in local osmotic pressure drop along the membrane chamber are comprehensively compared for FO and PRO modes. Finally, the capability of the CFD model to simulate a lab-scale PRO module is demonstrated across a range of Reynolds numbers from low-speed laminar up to turbulent flow regimes.

## 1. Introduction

Osmosis can be described as the spontaneous flow of water from a solution of high– water potential (low salinity) to a solution of low water potential (high salinity) across a semipermeable membrane [1]. Osmotic–driven processes play a significant role in modern engineering systems and industries, from separation and purification units to clean water and energy production plants [2,3,4]. There are three basic osmotic processes based on the water flow direction that depend on both the osmotic and hydraulic pressure difference: Reverse Osmosis (RO), Forward Osmosis (FO), and Pressure–Retarded Osmosis (PRO). In the FO process, there is no hydraulic pressure difference and hence the water flux is generated by the osmotic pressure difference only from the low-salinity (feed) solution to the high–salinity (draw) solution. In PRO, the hydraulic pressure difference applied to the draw solution is lower than the osmotic pressure difference, and, hence, water will still flow from the low–salinity to the high–salinity solution, so it is osmotic–driven like the FO process.

This class of membrane–based processes, i.e., PRO, has recently been investigated by several researchers due to its potential as a clean and sustainable energy source. However, there are limitations that are currently slowing down its scale–up and worldwide spreading [4]. Despite the technical challenges ahead, recent investments and promising innovations by start-up companies, e.g., SaltPower in Denmark, are paving the way to the realization of osmotic energy harvesting on an industrial scale [5]. Thus, research and development are still needed to improve osmotic power efficiency, and its commercial viability.

The fast increase in the experimental study of PRO has been accompanied by an increase in CFD modeling studies since 2010 [6]. Even though the pioneering CFD works on osmotic-driven flows focused only on FO process simulation [7,8], later works included the two–dimensional (2D) PRO channel [9], and both FO and PRO modes [10,11]. Nevertheless, only a few researchers took advantage of CFD to analyze local hydrodynamics and mass transfer inside three–dimensional (3D) PRO modules [12,13]. This is particularly due to challenges and difficulties to model the interaction of flow and mass transfer in all membrane compartments (feed, draw, and porous support layer) in a PRO module [6].

One of the main advantages of CFD is that geometry modifications can be implemented and tested more easily than physical prototypes [14,15]. Also, using CFD, one can model and study 3D complex geometries and multi–physics processes [16,17], which is impossible via the analytical approaches. Thus, a CFD modeling framework for PRO simulation can be considered as an efficient virtual lab for the simulation and analysis of different osmotic–driven processes. Despite this usefulness of CFD, there are some considerations that should be taken into account for the modeling of an osmotically driven membrane process.

The major challenge in using CFD to describe an osmotic-driven system lies in the implementation of the membrane boundary conditions: here, unlike in pressure–driven systems, internal concentration polarization (ICP) inside the membrane porous support layer reduces the effective osmotic driving force significantly, and this effect therefore has to be explicitly accounted for in the CFD model. Fundamentally, two approaches can be categorized to capture the ICP phenomena: (1) solving the governing differential equations over the porous zone, e.g., using Brinkman porous medium model, and (2) modeling the effect of the porous layer via the analytical representation of the osmotic flux–ICP relation.

The first approach resolves hydrodynamics and mass transfer in the porous layer [18]. Thus, it requires a fine computational mesh in the membrane zone and also determination of the permeability of the porous layer (κ). Due to such requirements, and because the characteristic length scale of the membrane support structure, *O* (10^−4^ m), is much smaller than the length scale of the membrane module, it is not viable to resolve the porous layer in a 3D CFD model. Alternatively, the solute profile within the support can be calculated from integrating the convection–diffusion equation between the active layer and feed channel [12]. This is the second approach that involves adoption of an analytical but complete model that includes both hydrodynamics and mass transfer. Previously, CFD validations [6,8,13], performed by comparing simulation outputs against experimental results, verified that the second approach is also a reasonable approximation due to the weak interaction between external flow and concentration polarization in the porous layer. Hence, in this work we adopt the second approach using an analytical ICP model for definition of the membrane boundary conditions (BCs) in CFD simulation, i.e., fully coupling the model to the CFD solver in such a way that the osmotic flux (as a BC for CFD solver) is calculated based on the local concentrations at the external membrane surfaces, which are resolved in return by the CFD solution of feed/draw channel flows.

On the other hand, the effect of external concentration polarization (ECP) on the draw-side (dilutive ECP or ECP_d_) and feed-side (concentrative ECP or ECP_c_) of the membrane are important in osmotic-driven processes, especially in the case of high–concentration solutions. Some earlier studies [8,19] used Sherwood number correlations for ECP modeling, while others ignored the local concentration variation or estimated it using simplified assumptions [12,13]. Such approaches are limited to special module geometries as they are based on the boundary layer similarity or other simplifications for calculation of local concentrations on the membrane boundaries with the feed and draw solution streams. In the current research, we adopted a more general approach, that is, ECP effects are resolved directly by CFD via implementing boundary layer (BL) mesh grids fine enough to capture thin concentration boundary layers. This approach can be executed without any limitation on geometry complexity and flow mode/regime and, as aforementioned, all CP effects are included in the simulation via two-way coupling between the membrane osmotic flux–ICP model, and the simultaneous CFD solution of feed/draw channel flows (ECPs) [20].

The methodology presented in this paper, was implemented by means of a powerful commercial CFD solver (Ansys^®^ Fluent, ANSYS, Inc., Canonsburg, PA, USA), which can be linked to a variety of modeling and meshing tools on Ansys Workbench Platform. For the efficient adjustment of membrane BCs, user–defined functions (UDFs) were programmed and coupled with the CFD solver. To the authors’ knowledge, there is no previously published work on the CFD simulation of 3D PRO membrane chambers (including all CP effects), or on the implementation and validation of the Ansys Fluent solver for osmotic–driven membrane process simulations, including a comparison between FO and PRO modes.

In this paper, after a description of the physics of the PRO process in the next section, the details of the modeling approach, including the PRO flux and RSF calculations as BCs for the CFD solver, are presented. Then, the results of CFD model validation against the reference experimental data are discussed for different scenarios and membrane parameters in both FO and PRO mode. Moreover, flow visualization and a comprehensive comparison of ECP/ICP contributions in local osmotic pressure drop along the membrane chamber are presented for both PRO and FO modes. Finally, the capability of the presented CFD model for the simulation of the lab–scale mixed–mode PRO module is demonstrated on a range of Re numbers from laminar to turbulent flow regimes.

## 2. Materials and Methods

**Figure 1:**
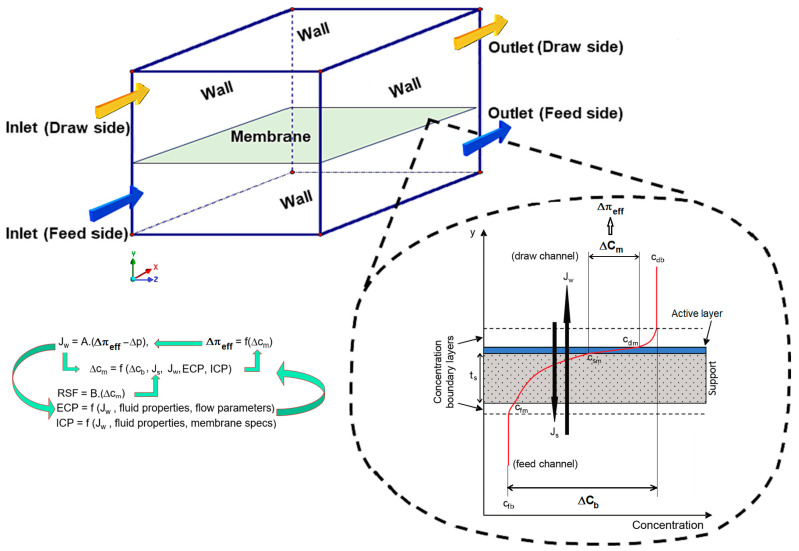
Schematic of a membrane module element (flat–sheet membrane in co–current flow mode) and cross–sectional details of the transmembrane mass transfer phenomena and local concentration profiles in PRO mode.

Osmotically driven membrane processes involve the movement of water through a semipermeable membrane due to an osmotic pressure difference. There are different key phenomena involved in transmembrane mass transfer in these processes. The driving force in such processes is the osmotic pressure difference between two solutions separated by a membrane. This pressure difference causes water to move from the side with lower concentration (feed solution) to the side with higher solute concentration (draw solution). The water flux across the membrane is influenced by the osmotic pressure gradient, the membrane properties, and the concentration polarization effect. So, in an osmotic process, there are different types of physics that have effect on the transmembrane flux and the system performance, including fluid dynamics, solute transfer, membrane separation, and membrane fouling, all of which are nonlinear and interdependent (see Figure 1).

There are also diverse scales of the involved phenomena in a membrane module. While the membrane active layer thickness is around 0.1 to 0.5 μm, the porous sublayer is in the range of 50 to 150 μm, whereas the module length can be from a few cm up to meters. Also, due to the fact that the concentration boundary layer is generally much thinner than the velocity boundary layer, high–fidelity modeling methods are required to capture the local concentration variations as the most important parameter in osmotically driven processes. As water moves through the membrane layers, solutes accumulate near the membrane surface on the feed side, creating a concentration polarization layer. This layer reduces the effective osmotic pressure difference and thus the osmotic water flux. As shown in Figure 1, there are both external and internal concentration polarization (ICP) phenomena involved in PRO. The external concentration polarization (ECP) stems from the diffusion boundary layer development near the dense layer of the membrane, but ICP occurs in the internal porous layer of the membrane. Managing concentration polarization is crucial for maintaining high efficiency in osmotic membrane systems. While ECP can be suppressed by increasing velocity or mixing, ICP is very difficult to mitigate.

In addition to osmotic water flux, there is also a reverse flux of solutes from the draw solution to the feed solution. This reverse solute flux can affect the overall efficiency and performance of the process. The extent of reverse solute flux depends on the membrane selectivity and the nature of the solutes. Therefore, for technical and economical feasibility studies of osmotic–driven systems, many laboratory and modeling works are required. Also, for efficient computational simulation of such multi–physics multi–scale processes, several factors should be taken into consideration in order to compromise between modeling accuracy and cost. In the following part of this section, we present details of the integrated framework developed in this study for simulation of osmotic–driven processes.

### 2.1. Governing Equations

The membrane module is simulated in an isothermal and steady–state condition. The governing equations for the conservation of mass, momentum, and solute mass fraction in a multi–species Newtonian fluid flow, neglecting the gravity effects, are

(1) $$\nabla \cdot \left(\rho u\right)=S_{m}\;\;,$$

(2) $$\nabla \cdot \left(\rho uu\right)=\nabla \cdot \left(\mu \left[\nabla u+\nabla u^{T}-\frac{2}{3}\left(\nabla \cdot u\right)I\right]\right)-\nabla P+S_{v}\;\;,$$

(3) $$\nabla \cdot \left(\rho um_{A}\right)=\nabla \cdot \left(\rho D_{AB}\nabla m_{A}\right)+S_{A}$$

where **u** is the fluid velocity vector, *S_m_*, *S_v_*, and *S_A_* are mass, momentum and solute source terms, *p* is the static pressure, *m_A_* is the local mass fraction of solute, and *D_AB_* is the solute diffusivity coefficient, which together with density (*ρ*) and dynamic viscosity (*μ*) denote the solution properties. Assuming a dilute aqueous solution of sodium chloride (NaCl), there are empirical correlations for the mixture properties [7]:

(4a) $$\rho =\rho_{w}+694m_{A}\;\;,$$

(5a) $$\mu =0.89\times 10^{-3}\left(1+1.63m_{A}\right)\;,$$

(6a) $$D_{AB}=max\;(1.61\times 10^{-9}\left(1-14m_{A}\right),\;1.45\times 10^{-9})$$

which are valid for NaCl solutions at 25 °C (*ρ_w_* = 997.1 kg/m^3^) for concentrations up to solute mass fractions of 0.09, corresponding to molar concentrations around 1.6 M [21]. All the case studies simulated in this work are iso–thermal (*T* = 25 °C), but, for different operational temperatures or non–isothermal conditions, we must implement more general empirical correlations, which also take into account the effect of temperature on the properties [10],

(4b) $$\rho =(-1.55c^{2}+45.5c+1123.3)exp(-0.004T)$$

(5b) μ=0.4599exp (0.10495c−0.021T)

(6b) $$D_{AB}=9.32\times 10^{-9}\exp\left(-2.63\times 10^{9}T^{-3.7}\right)\left(\begin{array}{c} 0.000182c^{5}-0.00172c^{4}-0.00142c^{3}\\ +0.0497c^{2}-0.0987c+1.0263 \end{array}\right)$$

where *c* (Mol/L) is molarity and *T* (K) is temperature. Also, the effect of pressure on the mixture properties is negligible for the operational pressures studied in this work.

The above system of equations can be solved numerically using a pressure–based CFD solver to determine the variations of velocity and concentration (*C* = *ρ m_A_*), in both the feed and draw channels, provided that we have laminar flow (${Re}_{H}=\frac{\rho ud_{H}}{\mu }$ < 2100) in both the feed and draw channels of the membrane chamber. In fact, for high–Reynolds–number flows, it is necessary to implement a turbulence model in the CFD simulation, where further parameters will be introduced depending on the turbulence modeling approach.

The simplest “complete models” of turbulence are the two–equation models in which the solution of two separate transport equations allows the turbulent velocity and length scales to be independently determined. One of the most popular two–equation models in practical engineering simulations is the k−ϵ model that has robustness, economy, and reasonable accuracy for a wide range of turbulent flows. The standard k−ϵ model is a semi–empirical model based on transport equations for the turbulence kinetic energy (*k*) and its dissipation rate (ϵ). As the strengths/weaknesses of the standard k−ϵ model have become known, improvements have been made to the model to improve its performance. One of these variants is the RNG k−ϵ model that we implemented for the turbulent case studies in this work. It is derived from the instantaneous Navier–Stokes equations, using a mathematical technique called “renormalization group” (RNG) methods [22].

The RNG k−ϵ model is similar in form to the standard k−ϵ, but includes some refinements and features that make it more accurate and reliable for a wider class of flows. Namely, in the derivation of the standard model, and the assumption is that the flow is fully turbulent, and the effects of molecular viscosity are negligible; it is therefore valid only for high–Reynolds–number flows, while the RNG k−ϵ model provides an analytically–derived differential formula for effective viscosity that accounts for low–Reynolds–number effects. For more details on the transport equations, the methods of calculating turbulent viscosity, and model constants, it is recommended to refer to the documentation of the CFD software (ANSYS Fluent) [22]. Specifically, here we just present a short discussion on the turbulent diffusivity calculation in the following.

If we are in the turbulent flow regime, and a turbulence model is on in the CFD model, then we need to take into account the turbulent diffusivity. The effective diffusivity is then equal to the sum of laminar diffusivity (*D_AB_*) and turbulent diffusivity (*D_turb_*). We know the laminar diffusivity from Equation (6), while the turbulent diffusivity can be computed from the turbulent Schmidt number (*Sc_turb_ = μ_turb_*/*rD_turb_*), where *μ_turb_* (the turbulent viscosity) is a function of turbulence parameters (k,ϵ). Hence, in turbulent flows, the mass diffusion is computed by replacing the mass diffusive flux term, i.e., $\left(\rho D_{AB}\nabla m_{A}\right)$ term in Equation (3), with $(\rho D_{AB}+\frac{\mu_{turb}}{{Sc}_{turb}})\nabla m_{A}$. In ANSYS Fluent, the turbulent Schmidt number is typically set to a user–specified constant (default value is 0.7, which is generally considered appropriate for most turbulent flows, as it reflects the empirical relationship between the turbulent transport of momentum and mass). However, in the RNG k−ϵ model, the RNG theory provides an analytical formula for turbulent Prandtl numbers, which is analogously used for turbulent Schmidt numbers, relating *Sc_turb_* and *Sc* (molecular Schmidt number) [22].

It must be noted that the specific turbulence model implemented in this work is just an example of several available turbulence models, and is not essentially the best candidate for this kind of simulation. It may be an interesting topic for future works to investigate and compare different turbulence models for the simulation of osmotic membrane modules. It is also noteworthy that, for both laminar and turbulent simulations, similar settings are used for the CFD solver, as shown in Table 1, except for the pressure–velocity coupling scheme, where the coupled solver is generally preferred for turbulent simulations due to its robustness and faster convergence, especially in cases involving complex geometries.

### 2.2. Boundary Conditions

In a PRO module, a thin semipermeable membrane structure separates the feed and draw channels with a dense selective (active) layer facing to the draw–side, and a porous support layer next to the feed–side of the membrane, as illustrated in Figure 1. Due to the concentration difference (Δ*C_m_*) between the two sides of the membrane, an osmotic–driven flow can be generated by the osmotic pressure difference (Δπ*_eff_*), which is always higher than the hydraulic pressure difference in the PRO case. So, the osmotic pressure, the osmotic water flux (*J_w_*), and reverse salt flux (*J_s_*) can be calculated via,

(7) $$\pi =(805.1\times 10^{5})\;m_{A},$$

(8) $$J_{w}=A\left(∆\pi_{eff}-∆P\right)\;,$$

(9) $$J_{s}=B∆C_{m}$$

where the proportionalities are the van’t Hoff correlation coefficient (805.1 bar), water permeability (*A*), and salt permeability (*B*), which are the intrinsic membrane properties, typically determined experimentally via RO tests [23].

The most important parameters in osmotic membrane modeling are *J_w_* and *J_s_*, which should be calculated via Equations (7)–(9) as BCs of the CFD solution. The flux equation can be solved either by having wall flux conditions that explicitly allow the matching flux pair to be set on either side of the membrane or by using a source–sink term pairing [18]. For the latter approach, it is crucial to note that the source at the membrane wall on the feed–side must have the opposite sign but same value as on the draw–side. Here, we are following the second approach that is considering the wall BC for the active layer and pairing source–sink terms for the adjacent cells on both sides of the membrane. A similar approach has been recently used for CFD simulation of RO membranes by Bae et al. [16], but here we modified and extended its formulation to the simulation of PRO membranes. Initially, all the source terms in the computational domain are zero. Next, in each CFD solver iteration, the PRO flux will be accounted for through mass/solute source–sink terms (*S_m_* and *S_A_* in Equations (1) and (3)), as they become non–zero in the mesh cells neighbored with the membrane walls, according to the following equations:

(10) $$S_{w}={\rho_{w}J}_{w}∆A_{m}/∆V_{m}\;\;,$$

(11) $$S_{A}=-J_{s}∆A_{m}/∆V_{m}\;,$$

(12) $$S_{m}=S_{w}+S_{A}$$

where the water and solute source terms (*S_w_*, *S_A_*) have dimensions of kg/(m^3^·s), Δ*V_m_* is the volume of a mesh cell directly in contact with the membrane wall, and Δ*A_m_* denotes the corresponding face area. It is notable that source terms can be defined for the momentum equation as well, i.e., *S_v_* in Equation (2); however, they are usually negligible owing to the small velocity in the boundary layer [18].

All the BCs for different boundaries in feed and draw channels are listed in Table 2. It is noteworthy that a complete 3D model is essential for capturing the full complexity of fluid flows in practical applications, but it is possible to reduce the computational domain using symmetry BC, if the geometry and fluid flow are symmetrical, which significantly decreases the computational resources and time required for simulations. However, while symmetry BCs can simplify 3D problems and make them more computationally feasible, the results obtained from 3D simulations with symmetry can still differ from purely 2D simulations, due to the inherent differences in dimensionality, 3D boundary layer effects, secondary flows, and turbulence modeling.

### 2.3. ICP Model

Recalling Equations (8) and (9), it can be seen that for the calculation of *J_w_* and *J_s_* we need the values of local concentrations (or salt mass fractions) on both sides of the membrane active layer, i.e., *Cd_m_* and *Cs_m_* (see Figure 1). *Cd_m_* is known from the CFD solution of the draw channel flow, but *Cs_m_* is determined via an analytical ICP model, which is a more efficient approach compared with the numerical solution of Brinkman equations governing the incompressible flow of the solution in the homogenous and isotropic porous support. Assuming *J_w_* as the dominant fluid velocity across the support layer in the PRO membranes, and neglecting the gradients in all directions except normal to the membrane surface, the Brinkman equations can be reduced to an ODE for solute mass balance in the porous layer:

(13) $$D_{s}\frac{dC\left(y\right)}{dy}-J_{w}C(y)=J_{s}$$

where *C*(*y*) is the concentration at position *y*, and $D_{s}=\varepsilon D_{AB}/\tau$ is the effective diffusivity of the solute at the support layer, whose porosity (*ε*) and tortuosity (*τ*) are known. By their definitions, *J_w_* and *J_s_* are defined as variables only at the position of the selective layer surface, i.e., *y* = *t_s_*. However, it has been shown that the fluxes derived at the selective layer surface are accurate quantitative approximations of the flux and flow in the three–dimensional geometry throughout the membrane structure [24]. Thus, at a steady state, the conservation of mass governing the solute transport in the support layer can be described as Equation (13), and can be integrated over the porous thickness with the relevant boundary conditions [*C*(*y* = 0) = *Cf_m_*, *C*(*y* = *t_s_*) = *Cs_m_*], and the solute concentration on the porous–side of the active layer (*Cs_m_*) comes out as,

(14) $$C_{sm}=\left(C_{fm}+J_{s}/J_{w}\right)e^{J_{w}K}-J_{s}/J_{w}$$

where *K* represents the solute resistivity in the support layer and is defined as *K* = *S*/*D*, where $S={\tau t}_{s}/\varepsilon$ is the structural parameter of the membrane, which, together with A and B (water and salt permeability), form the three main parameters displaying the properties of a membrane. Replacing Equation (9), i.e., $J_{s}^{PRO}=B∆C_{m}=B(C_{dm}-C_{sm})$, in Equation (14) results in,

(15) $$C_{sm}=\frac{{J_{w}C}_{fm}e^{J_{w}K}+BC_{dm}\left(e^{J_{w}K}-1\right)}{J_{w}+B(e^{J_{w}K}-1)}$$

(16) $${∆C}_{m}=\frac{C_{dm}-C_{fm}e^{J_{w}K}}{1+B(e^{J_{w}K}-1)/J_{w}}$$

where both relations depend on the local concentrations on the feed–side (*Cf_m_*) and draw–side (*Cd_m_*) of the membrane, which are known from the CFD solution of channel flows. Now, the PRO water flux equation can be derived using Equations (7), (8) and (15)

(17) $$J_{w}^{PRO}=A\left\{\pi_{dm}-f_{os}C_{sm}-\Delta P\right\}=A\left\{\pi_{dm}-f_{os}\frac{{J_{w}C}_{fm}e^{J_{w}K}+BC_{dm}\left(e^{J_{w}K}-1\right)}{J_{w}+B(e^{J_{w}K}-1)}-\Delta P\right\}$$

where the osmotic pressure on the draw–side of the membrane ($\pi_{dm}$) is a function of the local draw–side mass fraction of salt, which is known from the CFD solution, and *f_os_* (=805.1 × 10^5^/*ρ*) at the porous–side of the active layer. *f_os_* can be determined having *C_sm_* from Equation (15) and the local density (*ρ_sm_*) from a concentration–based version of Equation (4), as derived in Appendix A.

It is also described in Appendix A that a linear approximation of the relation between osmotic pressure and salt concentration can be formulated with a constant proportionality of ${\bar{f}}_{os}=769.1e2$, i.e., $\pi \approx {\bar{f}}_{os}C$, which results in $∆\pi_{eff}={\bar{f}}_{os}∆C_{m}$. Hence, via Equations (8) and (16), an alternative equation for *J_w_* can be obtained without the need to evaluate the local density in the porous layer (i.e., *ρ_sm_*), i.e., Equation (18), which is the water flux equation of the PRO process used by almost all the previously published modeling works. We present the derivation in details here to clarify the assumptions and simplifications that were not mentioned in the literature. So, now the PRO water flux equation (Equation (17) can be rewritten as,

(18) $$J_{w}^{PRO}=A\left\{{\bar{f}}_{os}\frac{C_{dm}-C_{fm}e^{J_{w}K}}{1+\frac{B}{J_{w}}\left(e^{J_{w}K}-1\right)}-\Delta P\right\}=A\left\{\frac{\pi_{dm}-\pi_{fm}e^{J_{w}K}}{1+\frac{B}{J_{w}}\left(e^{J_{w}K}-1\right)}-\Delta P\right\}$$

As will be discussed later, there is no remarkable difference between the CFD results coupling the solver either with Equation (17) or Equation (18) for the PRO cases studied in this work, where the concentrations are in the range of van’t Hoff assumption applicability (<100 g/L). The advantage of Equation (17) is that it can be used for the PRO membranes with higher draw solution concentrations (with a nonlinear osmotic pressure–concentration relation) as well. Also, for FO case, it can be shown that in a similar manner the famous FO flux equation, i.e., $J_{w}^{FO}=\frac{1}{K}ln\frac{B+A\pi_{dm}}{B+J_{w}+A\pi_{fm}}$ [25], can be written in an analogous form as (see Appendix B),

(19) $$J_{w}^{FO}=A\frac{\pi_{dm}-\pi_{fm}e^{J_{w}K}}{e^{J_{w}K}+\frac{B}{J_{w}}\left(e^{J_{w}K}-1\right)}$$

**Figure 2:**
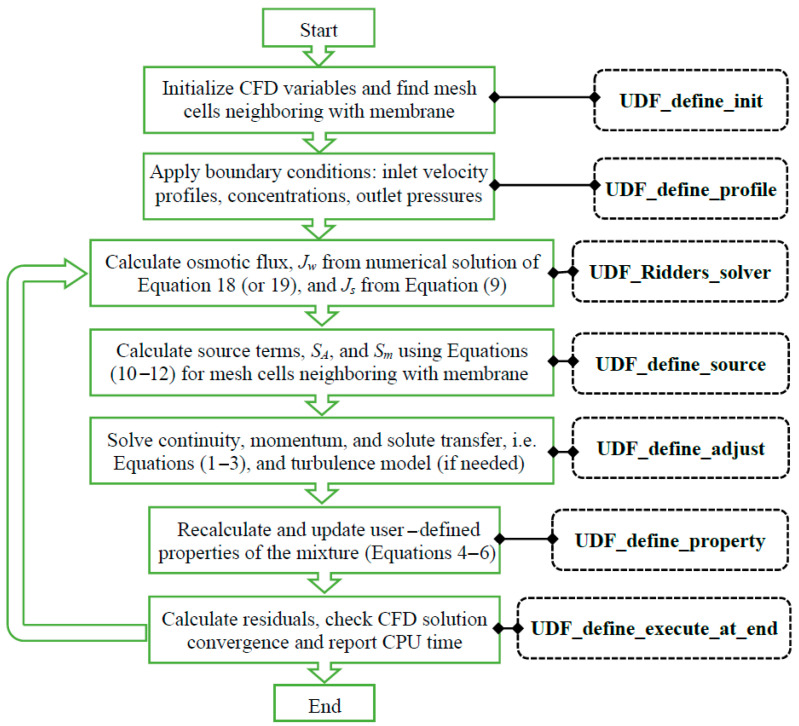
Flowchart of the numerical procedure of coupling membrane flux calculations to the CFD solver via user–defined functions.

Similarly, knowing the local values of $\pi_{dm}$ and $\pi_{fm}$ from the channel flow solution, or FO or PRO water flux equation, Equation (19) or Equation (18), will have only one unknown variable, i.e., *J_w_*. However, both equations are nonlinear and should be solved numerically (Figure 2) at each point on the membrane using a robust root–finding technique like Ridders’ solver, for which the details of its algorithm can be found in [26].

## 3. Results and Discussion

In this section, we present the results of different simulations carried out for the validation of the CFD model and demonstration of its capability to study 3D modules. All geometries were created using Ansys SpaceClaim, and Multizone meshes were generated by Ansys Meshing (version 2023R2, ANSYS, Inc., Canonsburg, PA, USA). All simulations were performed on a laptop computer (AMD Ryzen 9 4900HS with Radeon Graphics 3.00 GHz, 8-core/16-thread, installed with 32GB RAM). For each case study, several meshes were created for the mesh independence study, whose cell numbers depends on the module configuration and dimensions, e.g., the finest grid for the rectangular membrane chamber has ~1,500,000 cells.

### 3.1. Model Validation Using Rectangular Membrane Chamber (FO and Non–Pressurized PRO)

**Figure 3:**
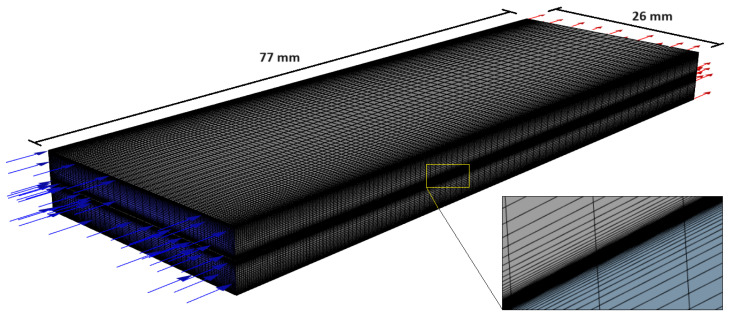
Membrane module geometry and the computational grid (including BL mesh) used for the validation of CFD model.

CFD model validation was carried out via the simulation of a rectangular co–current flow chamber, shown in Figure 3. The chamber was inspired by the one studied empirically in [23]. The area of the membrane segment was 20.02 cm^2^ (77 mm × 26 mm), located in the middle of the chamber. The height of both the feed and draw channels was 3 mm with no spacers in the channel. All flow parameters and membrane properties are summarized in Table 3. As seen in Table 3, two scenarios for feed and draw concentrations were examined to validate the CFD results. Also, two type of membranes were studied here in FO (active layer facing feed solution) and PRO (active layer facing draw solution) mode without a pressure difference between the feed and draw solutions (Δ*p* = 0). The membrane characteristics such as water permeability (A) and salt permeability (B) were as the average values of RO test data reported in [23] for the polyamide thin–film composite (TFC) membrane and also the cellulose triacetate asymmetric (CTA) membrane. For the CTA membrane, the thickness of the support layer (100 μm) and porosity/tortuosity ratio (ε/τ = 0.16) was obtained from [11], and for the TFC membrane the thickness of the support layer (50 μm) was estimated from the information in [27] and ε/τ = 0.24 was obtained from [28]. Due to the zero hydraulic pressure difference between the feed and draw channels, there is no compaction of the membrane support layer, so the membrane parameters are identical for both FO and PRO processes.

In CFD simulation of osmotic–driven processes, it is essential that the computational meshes are fine enough to capture all significant flow effects. So, the computational mesh here (Figure 3) consisted of boundary layer–type cells perpendicular to the membrane in both the feed and draw channel, graded such that the first grid points were located within 5 μm of the membrane in order to capture concentration polarization effects.

**Figure 4:**
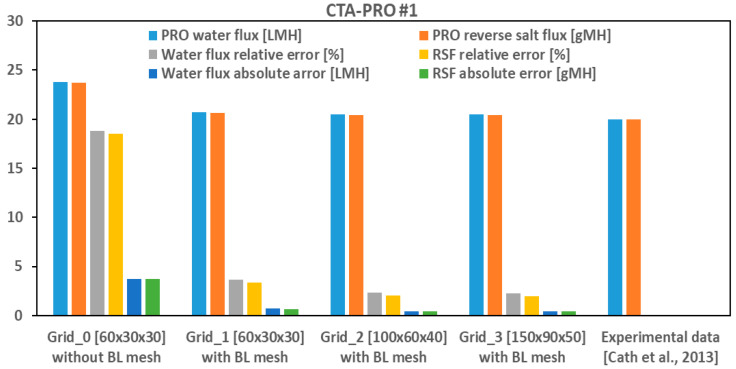
Grid study results of CFD model in comparison with experimental data [23].

As can be seen in Figure 4, a grid study was carried out starting from a coarse grid (Grid_0) without a BL mesh, then remeshing the same grid (~100 k cells) to include a BL mesh (Grid_1), and next increasing the grid size to 500 k (Grid_2) and 1500 k cells (Grid_3) to explore both the effects of a BL mesh and domain grid sizing on the PRO fluxes. It is observed in Table 4 that for Grid_0 (uniform mesh with first grid point within 100 μm of membrane surface) the results show about a 20% deviation from other plotted cases, while including a BL mesh in the same grid (non–uniform mesh with first grid point within 5 μm of the membrane surface) improves the accuracy of the predicted PRO fluxes. When we examined more refined domain meshes to ensure the grid independency of the results, it was revealed that increasing the number of domain cells (Grid_2, Grid_3) does not change the results much. Such an independency can be related to the non–uniform mesh sizing in the membrane chamber (finer mesh near the inlet/walls) that we used for all cases to capture sharp gradients of local osmotic fluxes near the inlet section and wall boundaries, which will be discussed later in the following sections.

Thus, it can be concluded that computational meshes of ~500,000 cells (e.g., Grid_2) are sufficient for this case, given that the grid points were scaled such that a fine BL mesh forms to capture a thin concentration boundary layer over the membrane surface. Similar norms are reported in [7,8] for similarly sized FO no–spacer chambers. It is also revealed from Figure 4 that there is excellent agreement between the CFD results and experimental data [23] for both the water flux and RSF of the studied case, i.e., CTA–PRO #1, even in the case of coarser grids, provided that a proper BL mesh is implemented. PRO fluxes were calculated by integrating the local fluxes (*J_w_*, *J_s_*) over all the cells locating on the membrane surface.

**Figure 5:**
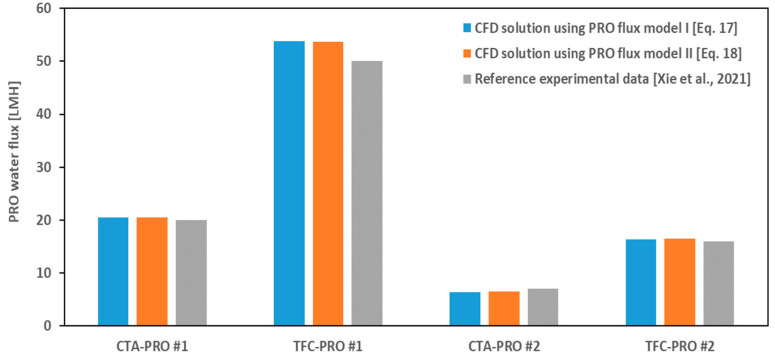
Comparison of osmotic water flux from simulations and the literature [11] in PRO mode.

For further validation of the model and to study the effect of feed/draw concentrations and membrane characteristics on the PRO flux, the results of the CFD simulations for the CTA and TFC membranes in scenarios #1 and #2 are presented in Figure 5. As seen in Figure 5, the PRO fluxes predicted by the proposed model, using either Equation (17) or Equation (18) for the flux calculation, are in agreement with the averaged experimental values reported in [11]. The maximum deviation is less than 10% and it is for the highest flux case (TFC–PRO #1), as the TFC membrane permeability is around ten times higher than the CTA, while its structural parameter is about one–third of the CTA. Of course, in practical PRO systems, the existence of a non–zero hydraulic pressure difference between feed and draw solution streams not only retards the rate of osmotic fluxes but also enforces utilizing thicker/denser porous layers (larger structural parameters) to enhance the mechanical stability of the membrane.

**Figure 6:**
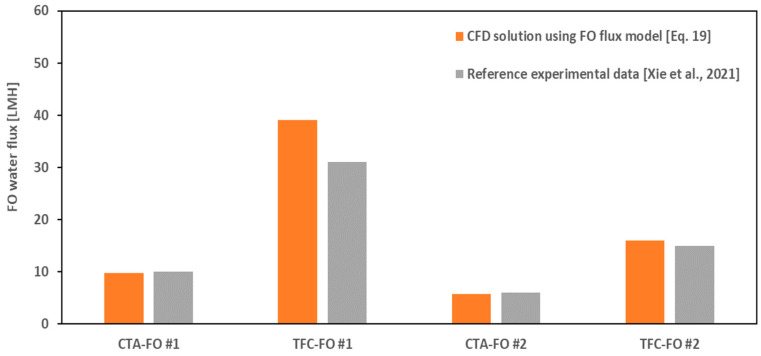
Comparison of osmotic water flux from simulations and literature [11] in FO mode.

To show the capability of the CFD model for handling different osmotic–driven processes, the results of simulations for both the CTA and TFC membranes in FO mode are presented in Figure 6 in comparison with the average values of the experimental data reported in [11]. The overall agreement between the CFD and lab data is good, as presented in Table 5, except for the case TFC–FO #1, where the water flux from simulation is ~30% higher flux than the lab data. This may be due to the increased complexity of ICP–flux nonlinear interactions in higher flux rates, which shows a need for a more accurate model than the classical flux equation (Equation (19)) for the FO case.

It can be also concluded from Figure 5 and Figure 6 that the presented CFD model has truly predicted the adverse effect of increased feed solution concentration on the osmotic flux. That is, for scenario #2 with higher feed/draw concentrations in comparison with scenario #1 but a similar bulk concentration difference (Δ*C_b_* = 1 M NaCl), the PRO and FO fluxes have been reduced by ~70% and ~50%, respectively. The same trend is reported and validated by [29] for a FO membrane under constant osmotic pressure difference, using various feed and draw solute concentrations. They developed an analytical FO flux model including all ICP, ECP, and RSF terms and showed that, even though the concentrative ECP on the feed–side of the membrane may be relatively insignificant when the feed solution is pure water, it is not negligible in a feed solution with high solute concentration, e.g., seawater. In addition, they concluded that higher a draw solute concentration caused severe ICP [29]. But here we showed (see Equation (15) for PRO mode and Equation (A6) for FO mode) that both the feed and draw concentration gradients (ECPd and ECPc) have influence on the value of the local concentration at porous–side of the active layer (*Cs_m_*), which determines the contribution of ICP in the total osmotic pressure drop across the membrane.

### 3.2. Visualization of Local Osmotic Fluxes and Concentration Polarization Effects (FO and PRO)

**Figure 7:**
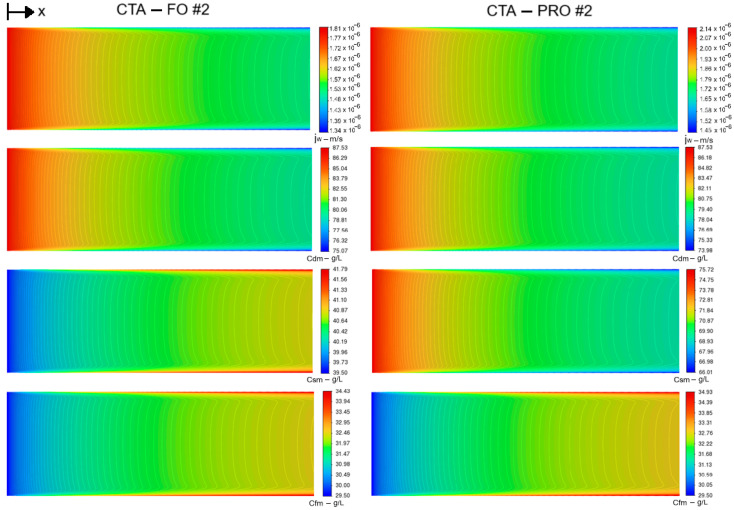
From top to bottom: contours of local flux (*J_w_*) and concentrations (*Cd_m_*, *Cs_m_*, *Cf_m_*) on the corresponding membrane surface.

CFD modeling offers the capability of local and interfacial visualization of variations over the entire membrane module, which is not possible by the experimental methods due to the limitations of the measurement tools and different scales of osmotic–driven flows. Here, for example, we have presented in Figure 7 the local water flux and concentration contours on the corresponding surfaces of CTA membrane in PRO and FO modes (scenario #2). As can be observed in Figure 7, highest osmotic fluxes occur near the inlet section where the local concentrations (*Cd_m_*, *Cf_m_*) on the membrane do not differ much from the entrance bulk values, but the local osmotic flux decreases gradually alongside the membrane towards the outlet, due to the osmotic pressure drops caused by CP effects, i.e., dilutive ECP on the draw–side, concentrative ECP on feed–side, and ICP inside of the membrane.

The intensity of the ECP effect, i.e., diffusion rate of water (or salt) passing through the membrane into the feed/draw mainstreams, depends on the rate of osmotic fluxes, and the flow mode/regime. For example, in our case of laminar co–current cross–flow, the contours of *J_w_* and *Cd_m_* show a similar declining trend towards the module outlet (see Figure 7). However, the minimum osmotic flux (highest osmotic pressure drop) occurs near the membrane–wall junctions, where the concentration boundary layer thickness is higher than the middle of channel. These observations are in agreement with the results of the previous CFD studies [19]. It is noteworthy that [19] implemented Sherwood number (*Sh*) correlations to account for the ECPd in the osmotic flux calculation, while in this study 3D concentration boundary layers on both sides of the membrane were resolved using fine CFD grids to obtain the accurate values of *Cd_m_* and *Cf_m_* for the osmotic flux calculation without the limitations of *Sh* correlations for 3D complex flows.

**Figure 8:**
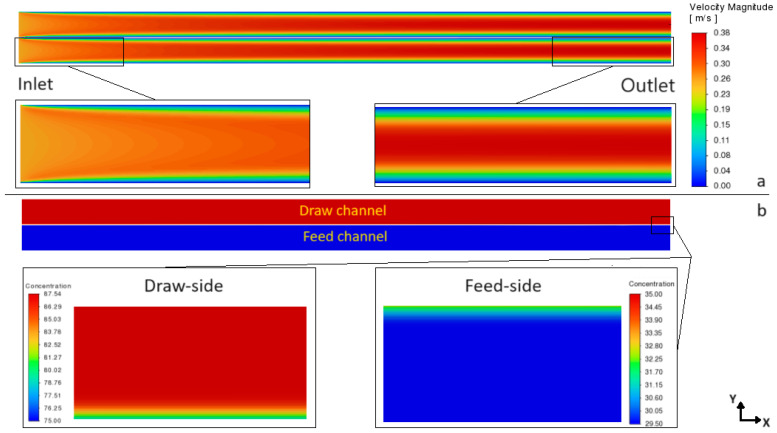
(**a**) Velocity contours and (**b**) concentration contours, at the middle longitudinal section of module in CTA–FO #2 case.

It is also noticeable from Figure 7 that, for both PRO and FO modes, the values of *Cf_m_* have similar growing trends along the membrane due to an increasing concentrative ECP effect, but for the local concentration on the porous–side of the active layer (*Cs_m_*) the opposite trends are observed in PRO and FO modes in this case study (CTA–FO #2). However, as will be discussed later in this section, this is not a typical trend and it can change in different scenarios due to the nonlinearity of ICP–flux dependency in FO mode and the significant impact of the feed concentration on the *Cs_m_* value (see Equation (A6) in Appendix B). Thus, using the current modeling approach, not only are the ECP_d_ and ECP_c_ effects directly captured by the CFD solution, but we can also study the 3D details of the velocity/concentration profiles over the entire membrane module, as shown in Figure 8, where the development of thin concentration boundary layers along with the thicker velocity ones are shown.

**Figure 9:**
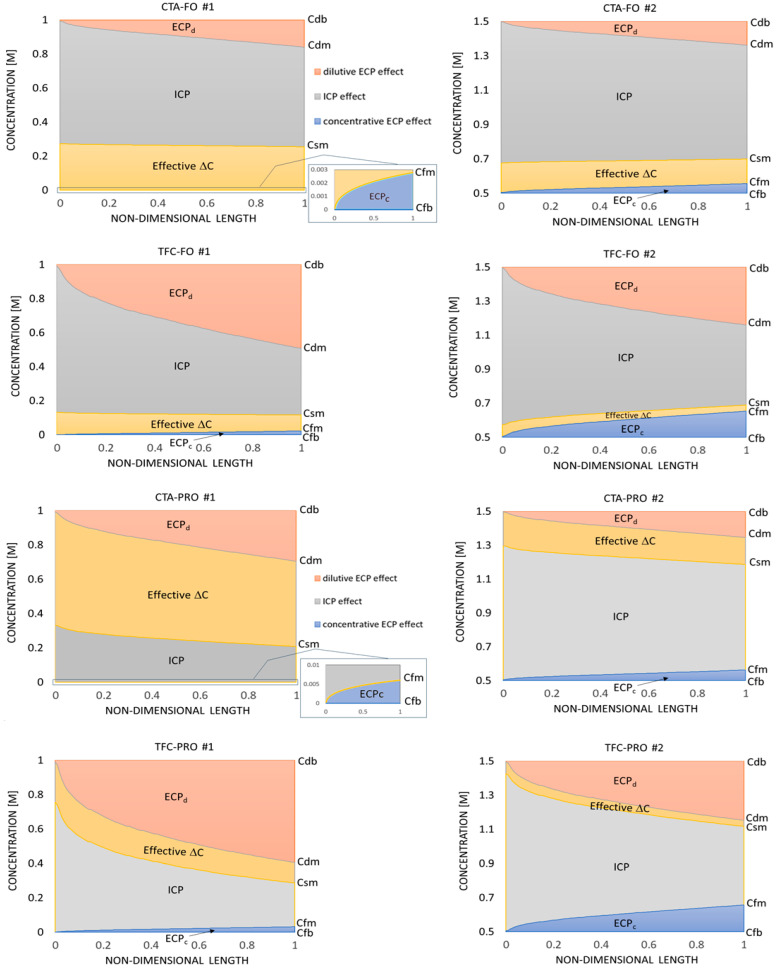
Variation in the sources of osmotic pressure drop along the membrane longitudinal centerline in FO and PRO (Δ*p* = 0).

To realize the contribution of various sources of osmotic pressure drop in different modes and scenarios, we compare in Figure 9 the variation in local concentrations (*Cd_m_*, *Cs_m_*, *Cf_m_*) along the membrane longitudinal centerline. It can be seen that for all cases, either in PRO or FO mode, the local effective concentration difference (or driving force) decreases from inlet to outlet, implying that the osmotic flux will be unchanged after a length enough for the membrane module to reach an equilibrium between the osmotic and bulk flows. It can be seen in Figure 9 that, for all scenarios, the ECP_d_/ECP_c_ contribution in the total osmotic pressure drop grows from inlet to outlet, while the ICP effect reduces along the membrane, regardless of the *Cs_m_* variation trend.

**Figure 10:**
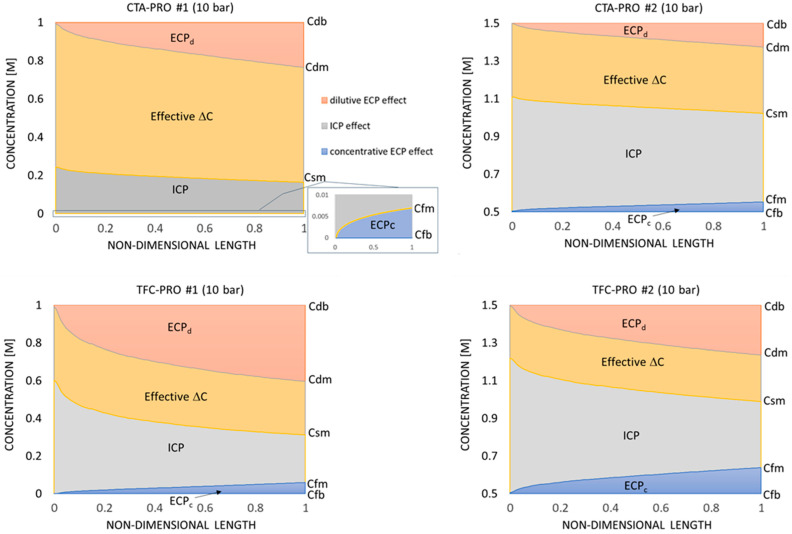
Variation in the sources of osmotic pressure drop along the membrane longitudinal centerline in PRO mode (Δ*p* = 10 bar).

The comparison between the results of scenario #1 and #2 in Figure 9, shows that a higher feed concentration in a FO/PRO system (with the same operating conditions) increases the total osmotic pressure drop and the corresponding ECPc/ICP contributions considerably. This adverse effect in PRO mode is more significant than FO mode. Also, the comparison between the results of CTA and TFC membranes proves that for a membrane with high permeability (lower structural parameter) all CP effects (ECP_d_, ECP_c_, and ICP) have greater contributions in the total osmotic pressure drop than the CTA membrane in similar scenarios. This difference is more evident in PRO mode. However, in a normal PRO (with Δ*p* ≠ 0), the sensitivity of the effective osmotic pressure to the membrane parameters is less (Figure 10).

Based on the results shown in Figure 9 and Figure 10, it can be concluded that, for a certain concentration difference between the feed and draw solutions (here, ΔC_b_ = 1 M NaCl), the more efficient way to reach a higher PRO flux is to use a low–concentrated feed solution, rather than utilizing a membrane with higher permeability, e.g., TFC membrane. This is because in the first approach only a higher ECP_d_ penalty will pay for the higher PRO flux, whereas in the latter all CP effects become more severe (see PRO cases in Figure 9 and Figure 10). This conclusion can be also confirmed if we compare the corresponding results in Figure 5.

The PRO process normally includes a certain hydraulic pressure difference (Δ*p*) between the feed and draw solution streams, e.g., utilizing high–pressure draw solutions. So, to see the impact of applying a pressure difference on the transmembrane CP effects, the same PRO scenarios are studied under Δ*p* = 10 bar. It is clear from Figure 10 that the overall trends of local concentrations (and local CP effects) for the new PRO scenarios are similar to the same cases with Δ*p* = 0 (Figure 9). However, the total osmotic pressure drop is less for the pressurized cases. This improved effective osmotic driving force can be related to the PRO flux reduction due to applying the hydraulic pressure difference (Equation (8)) and the consequent change in the local concentrations across the membrane via the ICP model (Equations (15)–(18)) and the source terms used in the CFD solution (Equations (10)–(12)). Hence, although the hydraulic pressure acts in the opposite direction to the osmotic pressure in PRO mode and causes reduction of the net osmotic flux, it may improve the membrane efficiency at the same time via reducing the CP effects. This “higher osmotic flux – higher osmotic pressure drop” rule of thumb can be compared with the familiar rule governing the fluid flow in pipes, i.e., for higher flux at a pipe outlet, higher hydraulic pressure loss should be overcome. Such a complex relation between the osmotic flux, the concentration (osmotic pressure) variations, and the membrane parameters in the osmotic–driven systems can be captured only by the modeling approach like the one presented in this paper.

### 3.3. CFD Simulation of Lab–Scale Membrane Module (PRO)

**Figure 11:**
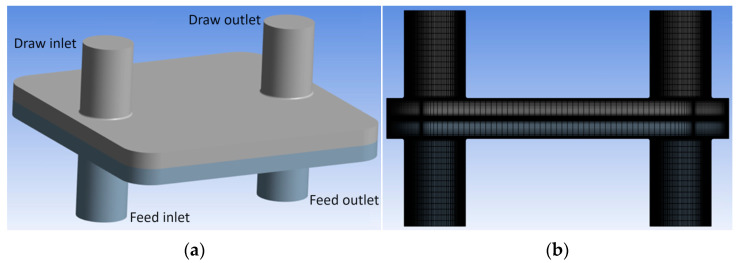
Lab–scale membrane module: (**a**) geometrical model and inlet/outlet configuration; and (**b**) center–plane cross–sectional view of the computational grid used for CFD simulation.

In a real membrane module, the nature of osmotic flow can be even more intricate, due to more complex geometries, mixed flow modes, and varied inlet configurations, which make the spatial variations of the osmotic flux and local concentrations more complicated. For a rectangular flow chamber, such as the one studied in previous sections, it is assumed that uniform streams enter the feed and draw channels and flow over the membrane surface (cross–flow) in the same direction (co–current mode) or opposite (counter–current mode). Nevertheless, for a real membrane chamber, e.g., the one showed in Figure 11, where the inlets are perpendicular to the cross–flow, it is not immediately apparent to what extent the chamber flow pattern is similar to the rectangular flow chamber. Some previous CFD studies [8] have modeled different lab–scale membrane modules to assess the accuracy of analytical film–theory ECP models for such membrane chambers in FO mode. However, using the CFD model presented in this paper, there is no limitation for modeling any kind of membrane module, as ECP effects are directly captured by the CFD solution.

Thus, in this section, the results of CFD simulation of a typical lab–scale membrane chamber (Figure 11) in PRO mode are presented. The chamber was inspired by a Sterlitech test module used commonly for RO tests [30]. The diameter of the inlet and outlet tubes is 7 mm and the length modeled here is 10 mm. The area of the membrane segment is 15.21 cm^2^ (39 mm × 39 mm) and the height of both feed and draw channels is 2.3 mm. The computational grid used for CFD solution has around 1 million cells including a BL mesh. All flow parameters and membrane properties are summarized in Table 6. To investigate the effect of different factors on the PRO flux, several simulations were carried out on a range of draw channel Reynolds numbers (Re_H_) from laminar to turbulent regime for different combinations of the membrane parameters (A, B, and S), as shown in Table 6. A(RO) and B(RO) denote water and salt permeability coefficients of a sample membrane determined from RO tests and it is assumed that we are free to change these parameters. Also, two values for the structural parameter of membrane are examined, 180 and 480 μm.

**Figure 12:**
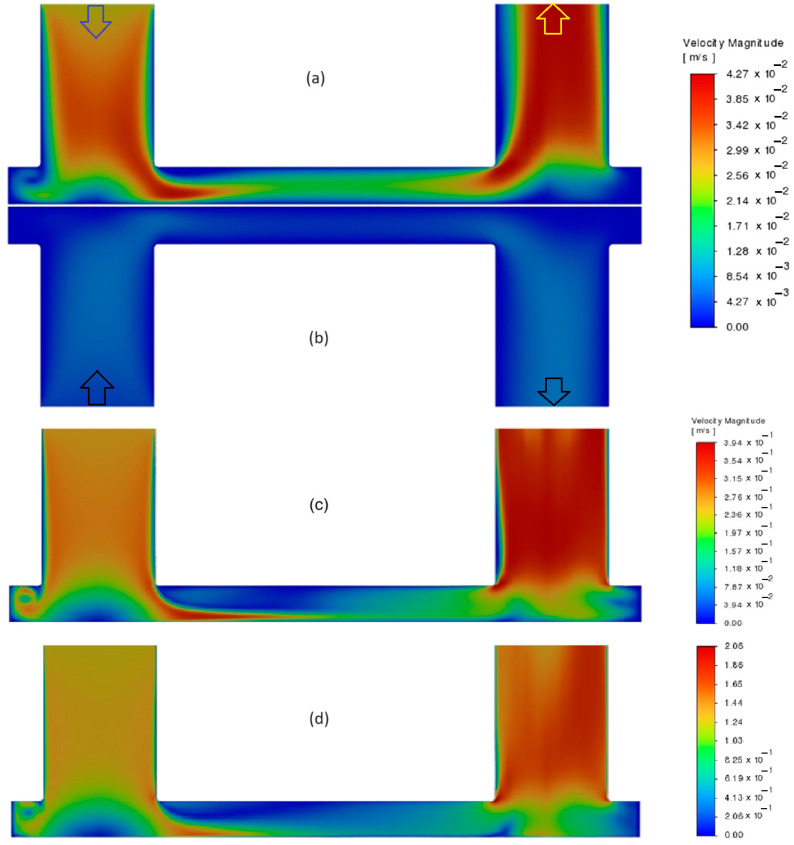
Velocity contours in a cross–sectional plane at middle of lab–scale membrane module: (**a**) draw channel, flowrate = 1.0 mL/s (Re_H_~50), (**b**) feed channel, flowrate = 0.17 mL/s (Re_H_~10), (**c**) draw channel, flowrate = 10 mL/s (Re_H_~500), (**d**) draw channel, flowrate = 50 mL/s (Re_H_~2500).

To focus on the most important CP effects in PRO mode, i.e., ECP_d_ and ICP, the feed channel flow regime was set to be a low–Re laminar flow for all cases (Figure 12). It can be seen in Figure 12 that when the inlet flow rate increases (as in draw channel here) the velocity gradients inside the chamber become broader and vortical especially near the contact area of the entrance flow and membrane. This happens due to the micro scales of the channel height and the perpendicular direction of the inlet flow with respect to the channel cross–flow on the membrane. Thus, the flow pattern varies over different parts of the membrane chamber, and so the validity of the analytical ECP models (*Sh* correlations) will be in doubt, as they assume cross–flow boundary layer similarity approximations for the ECP modeling [8].

**Figure 13:**
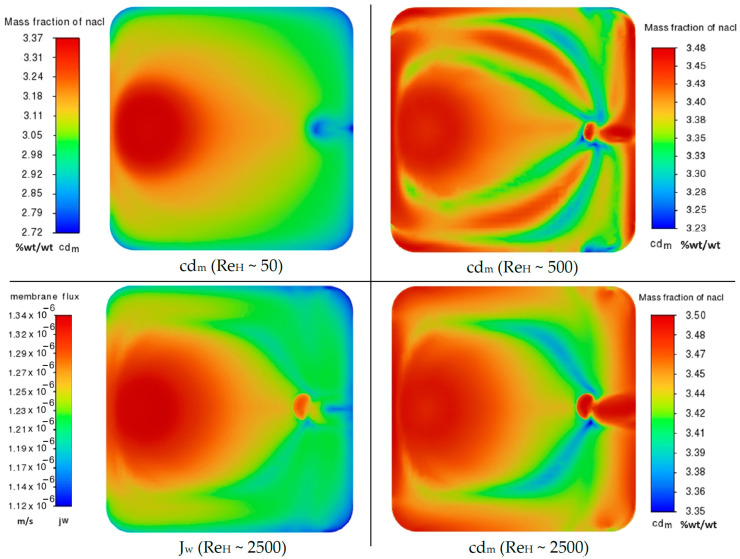
Contours of local salt mass fraction on the draw–side surface of the membrane (cd_m_), for Re_H_ = 50, 500, 2500; and contours of PRO flux for the turbulent case (Re_H_ = 2500).

Such a mixed–mode flow can be simulated only by the coupling of the osmotic flux model (including ICP) with a CFD solution of channel flows (resolving ECP), as presented in this paper. For example, in can be seen in Figure 13 that the contours of the salt mass fraction on the draw–side surface of the membrane in the laminar regime (Re_H_ ≈ 50) show a smooth transition from the max value (3.37%wt/wt) at the near–inlet area to the min value (2.72%wt/wt) at the dead zones of the membrane chamber, i.e., vicinity of walls and outlet region (see Figure 12). The stronger ECP effect near the walls and before the exit channel is due to the laminar boundary layer growth that is also observed in the case of the rectangular membrane chamber with Re_H_ ≈ 1300. However, for the lab–scale membrane chamber in Re_H_ ≈ 500, we can see a different pattern that demonstrates the important effect of fluid circulations (and mixing) on ECP_d_ reduction, even in the laminar regime: cd_m_ varies now between 3.48 and 3.23%wt/wt (Figure 13). The level of turbulence and mixing grows with the channel Reynolds number in such way that in the fully turbulent regime (Re_H_ ≈ 2500) there is no ECP_d_ effect on most areas of the membrane surface except some limited regions, as shown in Figure 13. In this case, the high inlet flux and rapid mixing of turbulent flow do not allow the osmotic water flux to dilute the local concentration of salt on the surface of the membrane, so cd_m_ has a value near the bulk inlet value, as it is in the range of 3.5–3.35%wt/wt for Re_H_ ≈ 2500 (see Figure 13).

**Figure 14:**
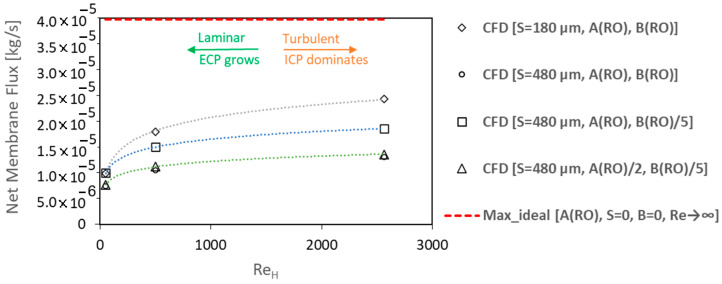
Effect of different membrane parameters on the trend of net membrane flux over a range of draw channel Reynolds numbers.

The contours of the local PRO flux for the turbulent case in Figure 13 reveal that, even with a negligible ECP_d_, the osmotic water flux can vary over the membrane due to ECP_c_ (and ICP) effects. However, in this case of high–Re draw channel flow, the variation is about ~16%. Thus, for the lab–scale membrane chamber working in the turbulent flow regime, the assumption of a uniform osmotic flux over the membrane surface can be a fair estimate. Also, the overall membrane flux will converge to a constant value as Re_H_ tends towards very high values (Figure 14). It is notable that if we could eliminate all ECP adverse effects, the maximum possible osmotic flux for a real membrane is still lower than the maximum ideal flux (without RSF and CP effects), due to ICP in the porous layer of the membrane. On the other hand, in low–speed laminar flows, the ECP_d_ growth can reduce the effective osmotic pressure and PRO flux in the same order of ICP, as shown in Figure 14.

It can be observed in Figure 14 that the trend of membrane flux versus Re_H_ is similar for all cases of membranes with different specifications (A, B, and S). Although, for the lower structural parameter case, the membrane flux is more sensitive to the cross–flow Reynolds number. In fact, the structural parameter has a great impact on the PRO flux, but for low–Re laminar flows its effect is nearly the same as the water permeability (A). Moreover, a 2x higher A value results in only ~30% added PRO flux for the highest Re_H_, which shows an increased ICP contribution in the osmotic pressure drop at a higher PRO flux. This reduction in membrane efficiency is similar to what was observed previously in the PRO case studies for the rectangular membrane chamber. In addition, it can be seen that a higher B value decreases the osmotic flux, but with a different order compared with the A value. Hence, the same flux results for the lab–scale membrane chamber (*S* = 480 μm) both with the original A and B (RO values) and with the reduced ones [A(RO)/2, B(RO)/5] values.

**Figure 15:**
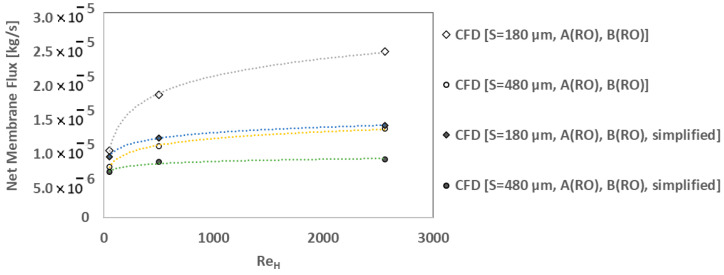
Effect of geometrical simplifications (neglecting the inlet–membrane flow interactions) on the net membrane flux trends.

It should be noted that, in low–Reynolds–number flows, the variation in osmotic water flux versus membrane properties may be similar to previous studies using 1D/2D simulations, especially for the simple membrane channel geometries with one dominant flow direction. However, for complex membrane chambers in real–world applications that include mixed–mode flow interactions, 3D boundary layer development, secondary flows and dead zones, accurate modeling of geometrical and fluid flow details is crucial for a reliable CFD simulation. For example, we have studied the lab–scale module assuming uniform flow through the device (neglecting fluid distribution through inlet/outlet tubes) to compare the results of a simplified model with the full model for two different structural parameters values. It can be observed in Figure 15 that the simplification of the lab–scale model not only deviates the osmotic flux trends substantially from the full model results, but also underestimates the effect of S and Re_H_ on the flux, especially in high Reynolds numbers. So, as shown in Figure 15, the relative changes (i.e., an increase or decrease in flux) is not the same for the full model and the simplified one when the mixing flow interactions and three–dimensional effects become stronger in higher inlet flow rates. In such conditions, simplified models or empirical correlations for mass transfer coefficients cannot represent the ECP effects accurately.

## 4. Conclusions

In this research, a unified CFD framework was developed and implemented for the 3D simulation of osmotically driven membrane processes, incorporating all concentration polarization effects (ECPd, ECPc, ICP) and reverse salt flux. The modeling methodology was presented in detail, based on a two–way coupling between the CFD solver and an analytical ICP model in FO or PRO mode. The model was validated against experimental data from a rectangular flow chamber under various operational scenarios, showing good agreement for the PRO fluxes, with a maximum deviation of less than 10%. Additionally, comparisons between different scenarios indicated that, for a constant inlet concentration difference, increasing feed/draw concentrations reduces the PRO and FO water fluxes by approximately 70% and 50%, respectively, in alignment with experimental observations.

CFD visualization of concentration profiles within the membrane chamber revealed that the highest osmotic fluxes are concentrated near the draw inlet section, whereas the minimum osmotic flux, corresponding to the greatest osmotic pressure drop, is observed near the membrane–wall intersections. Also, simulations of pressurized PRO modules demonstrate that the overall trends in local concentration profiles (and local CP effects) are similar with unpressurized cases (Δp = 0), even though the total osmotic pressure drop is lower in pressurized cases. A comparative analysis of the two membrane types indicates that, for membranes with higher water permeability (e.g., TFC), the CP effects contribute more substantially to the total osmotic pressure drop than for membranes with lower permeability (e.g., CTA), under analogous conditions. This disparity is more noticeable in PRO mode compared with FO mode. Consequently, it can be inferred that optimizing the PRO flux is more effectively achieved through the use of low–concentration feed solutions, rather than solely relying on membranes with high permeability.

CFD simulations of a lab–scale PRO chamber across a range of Reynolds numbers indicate that, under high–Re flow conditions, internal concentration polarization (ICP) is the dominant CP effect. Conversely, in low–velocity laminar flow regimes, the increase in external concentration polarization on the draw side (ECPd) can diminish the effective osmotic pressure and PRO flux to the same extent as ICP. In addition, analysis of various membrane parameter combinations indicates that the structural parameter (S) exerts a significant influence on PRO flux. However, under low–Re laminar conditions, its effect is nearly equivalent to that of water permeability (A). The influence of solute permeability coefficient (B) is several orders of magnitude lower.

In conclusion, the presented modeling framework exhibits sufficient flexibility for the simulation of 3D osmotically driven membrane modules with complex geometries, while also providing the capability to visualize local variations in various flow regimes. The proposed CFD model can be integrated with other solvers to conduct multi–physics simulations. Future research may explore dynamic membrane modules, fluid–structure interactions (FSI) in high–pressure membrane systems, hybrid models such as MD–CFD for including interphase interactions and microscopic inhomogeneities, and development of machine learning (ML) models to optimize osmotically driven membrane modules.

## Figures and Tables

**Table 1:** Methodology of the CFD solver for the simulation of osmotic–driven membranes.

Solver Mode	Viscous Model	Species Model	Pressure–Velocity Coupling
3D Parallel Double precision	Laminar/ RNG k−ϵ	Species transport	SIMPLE/ Coupled

**Table 2:** BCs for CFD simulation of osmotic–driven membrane module (see Figure 1).

Boundary	Mixture Fluid Flow		Solute Mass Transport	
	Feed Channel	Draw Channel	Feed Channel	Draw Channel
Inlet	Mass flow rate/velocity (*u_fb_*)	Mass flow rate/velocity (*u_db_*)	Concentration (*C_fb_*)	Concentration (*C_db_*)
Outlet	Static pressure (*P_fb_*)	Static pressure (*P_db_*)	Zero–gradient ($\frac{\partial C}{\partial n}=0$)	Zero–gradient ($\frac{\partial C}{\partial n}=0$)
Membrane	No–slip wall + mass sink (–*S_m_*)	No–slip wall + mass source (*S_m_*)	No–slip wall + solute source (–*S_A_*)	No–slip wall + solute sink (*S_A_*)
Walls	Impermeable wall (*u* = 0)	Impermeable wall (*u* = 0)	Impermeable wall ($\frac{\partial C}{\partial n}=0$)	Impermeable wall ($\frac{\partial C}{\partial n}=0$)

**Table 3:** Case study parameters for the validation of CFD simulations.

Parameter	Value	Units	Note
Feed/draw inlet velocity (flux)	0.25 (0.02)	m/s (L/s)	Re_H_ ≈ 1300 (d_H_ = 5.4 mm)
Feed/draw hydraulic pressure	<0.2 (3)	bar (psi)	Gauge pressure = 1 atm
Feed concentration—scenario #1	0.00	g/L	Deionized water
Draw concentration—scenario #1	58.44 (5.6)	g/L (wt/wt%)	=1.0 M NaCl
Feed concentration—scenario #2	29.22 (2.9)	g/L (wt/wt%)	=0.5 M NaCl
Draw concentration—scenario #2	87.66 (8.3)	g/L (wt/wt%)	=1.5 M NaCl
CTA membrane water permeability	2.36 × 10^−12^	m/s.Pa	=0.85 LMHbar
TFC membrane water permeability	2.42 × 10^−11^	m/s.Pa	=8.70 LMHbar
CTA membrane salt permeability	1.81 × 10^−7^	m/s	
TFC membrane salt permeability	2.52 × 10^−6^	m/s	

**Table 4:** Absolute and relative (%) errors of grid study cases shown in Figure 4.

Grid	Absolute Error (J_w_)	Absolute Error (J_s_)	Relative Error (J_w_)	Relative Error (J_s_)
Grid_0	3.76	3.70	18.8	18.5
Grid_1	0.73	0.67	3.65	3.35
Grid_2	0.47	0.41	2.35	2.05
Grid_3	0.46	0.40	2.30	2.00

**Table 5:** Absolute and relative (%) error of validation case studies shown in Figure 5 and Figure 6.

Case	Absolute Error (CTA)	Relative Error (CTA)	Absolute Error (TFC)	Relative Error (TFC)
PRO #1	0.47	2.35	3.75	7.50
PRO #2	0.50	7.14	0.49	3.06
FO #1	0.18	1.80	8.19	26.4
FO #2	0.26	4.33	1.05	7.01

**Table 6:** Case study parameters for CFD simulation of lab–scale membrane module.

Parameter	Value	Units	Note
Feed inlet mass flow rate	0.17	mL/s	Re_H_ ≈ 10 (d_H_ = 4.3 mm)
Draw inlet mass flow rate	1, 10, 50	mL/s	Re_H_ ≈ 50, 500, 2500
Feed outlet static pressure	5	bar	Δ*p* = 4 bar
Draw outlet static pressure	1	bar	Gauge pressure = 1 atm
Feed solution concentration	0.006 (0.0)	g/L (wt/wt%)	≈0.00 M NaCl
Draw solution concentration	36 (3.5)	g/L (wt/wt%)	=0.62 M NaCl
Membrane water permeability	0.4–4.0	LMHbar	A(RO) = 4 LMHbar
Membrane solute permeability	0.4–2.0	LMH	B(RO) = 2 LMH
Membrane structural parameter	180,480	μm	

**Figure A1:**
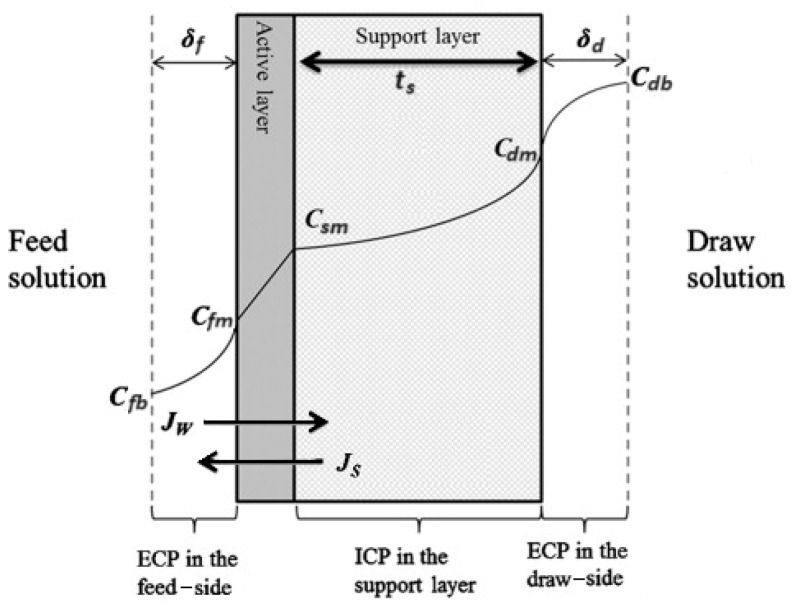
Concentration profiles across the membrane layers in FO mode.

## Data Availability

Data will be made available on request.

## Nomenclature

***Abbreviations***		*c*	*Concentration or Molarity* [mol/L]
BC	*Boundary condition*	*d_H_*	*Channel hydraulic diameter* [mm]
BL	*Boundary layer*	*D_AB_*	*Solute diffusivity* [m^2^/s]
CFD	*Computational Fluid Dynamics*	*D_s_*	*Effective solute diffusivity* [m^2^/s]
CP	*Concentration polarization*	*D_turb_*	*Turbulent diffusivity* [m^2^/s]
CTA	*Cellulose triacetate asymmetric*	*f_os_*	*Osmotic pressure–concent. ratio* [m^2^/s^2^]
ECP_d_	*External concentration polarization* *(dilutive)*	*J_s_* *J_w_*	*Reverse salt flux* [kg/s.m^2^] [gMH] *Osmotic water flux* [m/s] [LMH]
ECP_c_	*External concentration polarization* *(conservative)*	*k* *K*	*Turbulence kinetic energy* [m^2^/s^2^] *Solute resistivity of support layer* [s/m]
FO	*Forward Osmosis*	*m_A_*	*Local mass fraction of solute* [kg/kg]
gMH	g.m^−2^.h^−1^	*p*	*Static pressure* [Pa] [bar]
ICP	*Internal concentration polarization*	Δ*p*	*Applied hydraulic pressure difference*
LMH	L.m^−2^.h^−1^	*Re_H_*	*Channel Reynolds number*
LMHbar	L.m^−2^.h^−1^.bar^−1^	*S*	*Structural parameter* [μm]
ODE	*Ordinary Differential Equation*	*S_A_*	*Solute source term* [kg/(s.m^3^)]
PRO	*Pressure–Retarded Osmosis*	*S_m_*	*Mass source term* [kg/(s.m^3^)]
RANS	*Reynolds–Averaged Navier–Stokes*	*S_v_*	*Momentum source term* [kg/(s^2^.m^2^)]
RNG	*Renormalization group*	*S_w_*	*Water source term* [kg/(s.m^3^)]
RO	*Reverse Osmosis*	*Sc*	*Molecular Schmidt number*
RSF	*Reverse salt flux*	*Sc_turb_*	*Turbulent Schmidt number*
SIMPLE	*Semi–Implicit Method for Pressure Linked Equations*	*t_s_* *u*	*Membrane thickness* [μm] *Fluid velocity vector* [m/s]
TFC UDF	*Thin–film composite membranes* *User–defined function*	Δ*V_m_*	*Volume of mesh cells directly in contact with membrane boundary walls* [m^3^]
***Symbols***		***Greek letters***	
A	*Water permeability* [m/s.Pa] [LMHbar]	ϵ	*Turbulence dissipation rate* [m^2^/s^3^]
Δ*A_m_*	*Face area of mesh cells directly in contact with membrane boundary walls* [m^2^]	*ε*	*Porosity of membrane support layer*
		*μ*	*Dynamic viscosity* [Pa.s]
*B*	*Salt permeability* [m/s] [LMH]	*μ_turb_*	*Turbulent viscosity* [Pa.s]
*C*	*Concentration* [g/L]	*π*	*Osmotic pressure* [bar]
Δ*C_b_*	*Bulk flow concentration difference* [g/L]	Δ*π_b_*	*Bulk flow osmotic pressure difference*
Δ*C_m_*	*Effective concentration difference* [g/L]	Δ*π_eff_*	*Effective osmotic pressure* [bar]
*C_db_*	*Bulk draw flow concentration* [g/L]	*π_db_*	*Bulk draw flow osmotic pressure* [bar]
*C_dm_*	*Local concentration on the draw–side of membrane* [g/L]	*π_dm_*	*Local osmotic pressure on the draw–side of membrane *[bar]
*C_fb_*	*Bulk feed flow concentration* [g/L]	*π_fb_*	*Bulk feed flow osmotic pressure* [bar]
*C_fm_*	*Local concentration on the feed–side of membrane* [g/L]	*π_fm_*	*Local osmotic pressure on feed–side of membrane* [Pa] [bar]
*C_sm_*	*Local concentration on the support–side of membrane active layer* [g/L]	*ρ *	*Density* [kg/m^3^]
		*τ*	*Tortuosity of membrane*

## Appendix A

The correlation of density–mass fraction dependency for a mixture of salt–water [21], i.e., *ρ* = *ρ*_w_ + 694*m_A_*, can be reformulated by replacing *m_A_* with *C*/*ρ* to connect the density and concentration variables. Solving the resulting equation for *ρ* yields,

(A1) $$\rho =\frac{\rho_{w}}{2}+\sqrt{{\left(\frac{\rho_{w}}{2}\right)}^{2}+694C}$$

where *ρ_w_* = 997.1 kg/m^3^ is the fresh water density. Now for example, the local value of density at the porous–side of the active layer (*ρ_sm_*) can be determined based on the local concentration (*C_sm_*), which is associated to *C_dm_* and *C_fm_* via Equation (15). Therefore, we can formulate a relation between *ρ_sm_* and *J_w_* using Equations (15) and (A1),

(A2) $$\rho_{sm}=\frac{\rho_{w}}{2}+\sqrt{{\left(\frac{\rho_{w}}{2}\right)}^{2}+694\frac{{J_{w}C}_{fm}e^{J_{w}K}+BC_{dm}\left(e^{J_{w}K}-1\right)}{J_{w}+B(e^{J_{w}K}-1)}}$$

that can be used to make the PRO flux equation (Equation (17)) a single–unknown (*J_w_*) equation. Similarly, for the osmotic pressure calculations from the van’t Hoff correlation, i.e., Equation (7), replacing *m_A_* with *C*/*ρ*, and using Equation (A1), we can derive a direct relation between *π* and *C* as follows,

(A3) $$\pi =\frac{\left(805.1\times 10^{5}\right)C}{\rho }=\frac{\left(805.1\times 10^{5}\right)C}{\rho_{w}/2+\sqrt{{\left(\rho_{w}/2\right)}^{2}+694C}}\;$$

where, for *C* < 100 g/L (*m_A_* < 0.09 kg/kg), Equation (A3) can be approximated by the following linear equation (R–squared value = 0.9999),

(A4) $$\pi \approx \left(769.1\times 10^{2}\right)C={\bar{f}}_{os}C\;$$

where ${\bar{f}}_{os}$ is linear proportionality factor between the osmotic pressure and concentration.

## Appendix B

For a membrane in FO mode (active layer facing feed solution, as shown in Figure A1), similar to PRO mode, the steady–state equation of conservation of mass governing the solute transport in the support layer can be integrated over the porous thickness with the relevant boundary conditions [*C*(*y* = 0) = *Cs_m_*, *C*(*y* = *t_s_*) = *Cd_m_*], and the solute concentration at the porous–side of the active layer (*Cs_m_*) comes out as,

(A5) $$C_{sm}=\left(C_{dm}+J_{s}/J_{w}\right)e^{-J_{w}K}-J_{s}/J_{w}$$

where *K* represents the solute resistivity in the support layer, and *K* = *t_s_/D_s_*, where $D_{s}=\varepsilon D_{AB}/\tau$ is the effective diffusivity of the solute in water.

Replacing $J_{s}^{FO}=B∆C_{m}=B(C_{sm}-C_{fm})$ in Equation (A5) results in,

(A6) $$C_{sm}=\frac{{J_{w}C}_{dm}+BC_{fm}\left(e^{J_{w}K}-1\right)}{J_{w}e^{J_{w}K}+B(e^{J_{w}K}-1)}$$

and

(A7) $${∆C}_{m}=\frac{C_{dm}-C_{fm}e^{J_{w}K}}{e^{J_{w}K}+B(e^{J_{w}K}-1)/J_{w}}$$

Assuming a linear relation between the osmotic pressure and salt concentration, i.e., $\pi \approx {\bar{f}}_{os}C$, and $∆\pi_{eff}={\bar{f}}_{os}∆C_{m}$, and using Equations (8), (9) and (A7), the FO equations for the water and reverse salt fluxes (*J_w_* and *J_s_*) can be obtained as following:

(A8) $$J_{w}^{FO}=A\left\{{\bar{f}}_{os}\frac{C_{dm}-C_{fm}e^{J_{w}K}}{e^{J_{w}K}+\frac{B}{J_{w}}\left(e^{J_{w}K}-1\right)}\right\}=A\left\{\frac{\pi_{dm}-\pi_{fm}e^{J_{w}K}}{e^{J_{w}K}+\frac{B}{J_{w}}\left(e^{J_{w}K}-1\right)}\right\}$$

(A9) $$J_{s}^{FO}=B\left\{\frac{C_{dm}-C_{fm}e^{J_{w}K}}{e^{J_{w}K}+\frac{B}{J_{w}}\left(e^{J_{w}K}-1\right)}\right\}=\frac{B}{{\bar{f}}_{os}A}J_{w}^{FO}$$

where ${\bar{f}}_{os}=769.1\times 10^{2}$, for *C* < 100 g/L (1.6 M), as derived in Appendix A. Comparing FO flux equation (Equation (A8)) and PRO flux equation (Equation (18)) shows that they are almost similar except for the added exponential term in the denominator of Equation (A8). Hence, for similar flow conditions, i.e., same feed/draw concentrations and Δ*p* = 0, the osmotic membrane flux in PRO mode will be always larger than when the system is operating in the FO configuration.
